# Spin Transport in Organic Molecules

**DOI:** 10.3389/fchem.2019.00428

**Published:** 2019-06-18

**Authors:** Lidan Guo, Yang Qin, Xianrong Gu, Xiangwei Zhu, Qiong Zhou, Xiangnan Sun

**Affiliations:** ^1^Key Laboratory of Nanosystem and Hierarchical Fabrication, National Center for Nanoscience and Technology, CAS (Chinese Academy of Sciences) Center for Excellence in Nanoscience, Beijing, China; ^2^Center of Materials Science and Optoelectronics Engineering, University of Chinese Academy of Sciences, Beijing, China; ^3^Department of Materials Science and Engineering, College of New Energy and Materials, China University of Petroleum Beijing, Beijing, China

**Keywords:** molecular spintronics, molecular spin valve, spin transport, functional molecules, supramolecules

## Abstract

Because of the considerable advantages of functional molecules as well as supramolecules, such as the low cost, light weight, flexibility, and large area preparation via the solution method, molecular electronics has grown into an active and rapidly developing research field over the past few decades. Beyond those well-known advantages, a very long spin relaxation time of π-conjugated molecules, due to the weak spin-orbit coupling, facilitates a pioneering but fast-growing research field, known as molecular spintronics. Recently, a series of sustained progresses have been achieved with various π-conjugated molecular matrixes where spin transport is undoubtedly an important point for the spin physical process and multifunctional applications. Currently, most studies on spin transport are carried out with a molecule-based spin valve, which shows a typical geometry with a thin-film molecular layer sandwiched between two ferromagnetic electrodes. In such a device, the spin transport process has been demonstrated to have a close correlation with spin relaxation time and charge carrier mobility of π-conjugated molecules. In this review, the recent advances of spin transport in these two aspects have been systematically summarized. Particularly, spin transport in π-conjugated molecular materials, considered as promising for spintronics development, have also been highlighted, including molecular single crystal, cocrystal, solid solution as well as other highly ordered supramolecular structures.

## Introduction

The spin degree of freedom of electrons exhibits particular potential on information non-volatile memory, transport, and processing (Wolf et al., 2001). The π-conjugated molecules, usually composed of elements with low atomic numbers, theoretically possess extremely long spin relaxation times and thus excellent spin transport properties. Organic spintronics has thus attracted extensive attention in the past decade (Dediu et al., 2009; Sanvito, 2011) after the successful spin injection in molecular materials was first reported (Dediu et al., 2002). Additionally, abundant chemical tailorability, functionality, and mechanical flexibility of organic semiconductors (OSCs) give organic spintronic devices various accessional functionalities except for a spin valve effect, such as a light embittering, photovoltaic effect, or a photoresponse, etc (Guo et al., 2019). Undoubtedly, spin transport, an important and basic topic of organic spintronics, plays a crucial role in understanding the mechanism of spin-related phenomena and in developing novel multipurpose spintronic devices (Sun X. et al., 2016; Sun et al., 2017).

In a molecule-based spintronic device, spin-polarized carriers are normally injected from a magnetic electrode, and transported in the OSC, and are finally detected by another magnetic electrode before their spin polarization becomes totally relaxed (Jang and Richter, 2017). Thus, the spin injection efficiency and the spin transport process co-determines the spin transport distance, wherein, an efficient spin-polarized injection is a prerequisite of spin transporting in the molecular layer (Shim et al., 2008; Raman et al., 2009). In order to enhance the spin injection efficiency, ferromagnetic (FM) electrodes with high spin polarization and Curie temperature are preferably used. In particular, LSMO possesses 100% spin polarization but can only be realized at 4.2 K (Bowen et al., 2003), which greatly reduces its application potential. In contrast, Fe_3_O_4_ (Zhang et al., 2014), NiFe (Sun X. et al., 2016), Ni (Starko-Bowes et al., 2013), and Co (Sun et al., 2013) with high Curie temperature is more inclined to be used in the devices in room-temperature operations. In addition, a thin buffer layer is normally inserted at the FM/molecule interface to relieve the Schottky barrier or energy level mismatch, which has been demonstrated as a very efficient method to enhance spin injection. Layer materials include metallic oxides or fluorides [e.g., AlO_x_ (Sun et al., 2013; Zhang X. et al., 2013), MgO (Szulczewski et al., 2009), LiF (Schulz et al., 2011; Nguyen et al., 2012)], molecules (Prieto-Ruiz et al., 2019), self-assembled monolayers (Pookpanratana et al., 2015), etc. The spin-polarized carriers tunnel across such an interface and thus the problem of energy level mismatch can be ignored. It is also worth noting that the inserted interfacial layer on the top of molecular layer can also reduce the penetration from the top electrode, which will efficiently protect the molecular layer and facilitate the formation of spin valves rather than tunnel junctions (whose spin transport mode is tunneling due to very thin molecular materials). Furthermore, novel spin injection methods via a spin pumping process (Ando et al., 2013; Jiang et al., 2015; Sun D. et al., 2016) or via hot spin electron emitting (Appelbaum et al., 2007; Gobbi et al., 2012) are also being actively explored. Indeed, a few recent reviews have made a relatively detailed introduction to spin injection and spinterface (Cinchetti et al., 2017; Jang and Richter, 2017; Sun and Mi, 2018), which can be referenced as well.

**Figure 1:**
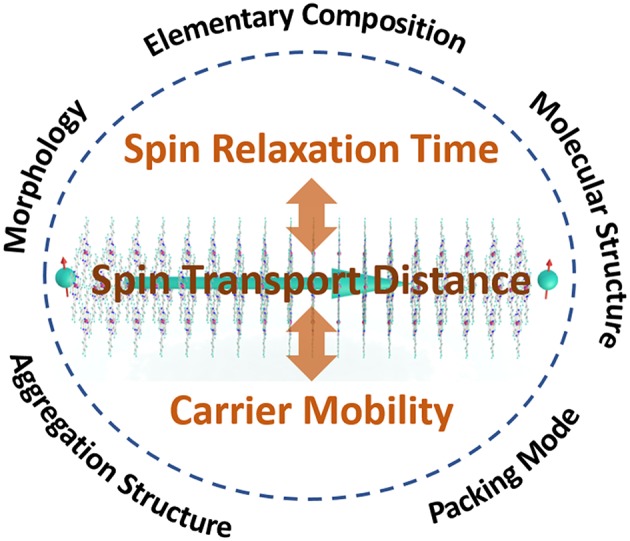
An overview of this review, with the key points of achieving long spin transport distances in OSCs, where spin relaxation time and mobility are the direct impacts affected by elementary composition, molecular structure, packing mode, aggregation structure, and morphology of OSCs.

The influence factors of spin transport performance in OSC can be found in Einstein's relationship, λ_*s*_ = $\sqrt{\left(D_{hop}+D_{ex}\right)\;{\;\tau }_{s}}$, where D_*hop*_, D_*ex*_, τ_*s*_, are the spin diffusion constants based on the hopping spin transport mode and exchange coupling mode, spin relaxation time of molecules, respectively. It is worth noting that, only with a high carrier concentration in the molecular layer, generally induced via the impurity band (Yu, 2014; Bergenti et al., 2018; Riminucci et al., 2019) or doped molecular semiconductors (Wang et al., 2019), is D_*ex*_ a prominent value and should therefore be seriously considered. An exchange coupling model provides a particularly fast speed compared to hopping transport since the spin transport process can decouple with charge transport. Moreover, the absence of the Hanle effect (Grünewald et al., 2013; Riminucci et al., 2019) and the low operating voltage (Barraud et al., 2010; Grünewald et al., 2014) in a device based on a molecular layer with an impurity band can also be explained. For the hopping transport mode, *D*_*hop*_=*k*_*B*_*Tμ*/*e*, where k_*B*_, μ are the Boltzmann constant, and charge carrier transport mobility, respectively. Therefore, from the Einstein relationship, a long spin relaxation time and high carrier concentration or mobility of OSC appears to improve the spin transport distance. The spin relaxation time therefore determines the duration of the spin polarization in OSC, which is mainly influenced by elemental compositions, molecular structure sand morphologies of OSC. The mobility of the semiconductor determines the transport rapid of spin-polarized carriers and is influenced by the molecular structures, packing mode, aggregation structure, as well as defects (Dong et al., 2013) (Figure 1).

One main purpose of this review is to provide guidelines for key points toward pursuing longer spin transport distances in OSCs, while the relationship between the characteristic of π-conjugated molecules and the spin transport process will be the focal point. First, by introducing two typical spin relaxation mechanisms, spin-orbit coupling (SOC) and hyperfine interaction (HFI), a systematic illustration on how the elementary composition and molecular structure affects the spin relaxation time will be provided. Second, has been found that the aggregation structure has dual effects on both the spin relaxation time and mobility. Thus, spin transport properties of OSCs, with three different aggregation structures including polycrystalline/amorphous thin films, single crystals as well as supramolecular assembly structures, are discussed and prospected, respectively. Additionally, a conclusion and outlook section has also been provided at the end in order to summarize the current achievements and outline challenges for obtaining longer spin transport distances.

## Spin Relaxation Mechanism in π-Conjugated Molecules

The SOC and HFI are considered to be the principal factors that cause spin relaxation in OSCs. So far, few studies have revealed that the modification of the elementary composition and chemical structure of molecules are feasible ways to weaken the SOC or HFI effects, therefore prolonging the spin relaxation time.

### Spin-Orbit Coupling

The SOC is the interaction between the spin and charge's orbital angular momentum, which has a double impact on spin devices: it contributes to the spin-charge conversion and also causes spin relaxation (Schott et al., 2017). Generally, the SOC strength is proportional to the fourth power of the atomic number, and the light-weight-element composition (such as C, H, O, N) of OSCs just leads to a very weak SOC strength and therefore extremely long spin relaxation time up to a millisecond level experimentally and even second level in theory (Pramanik et al., 2007; Watanabe et al., 2014).

**Figure 2:**
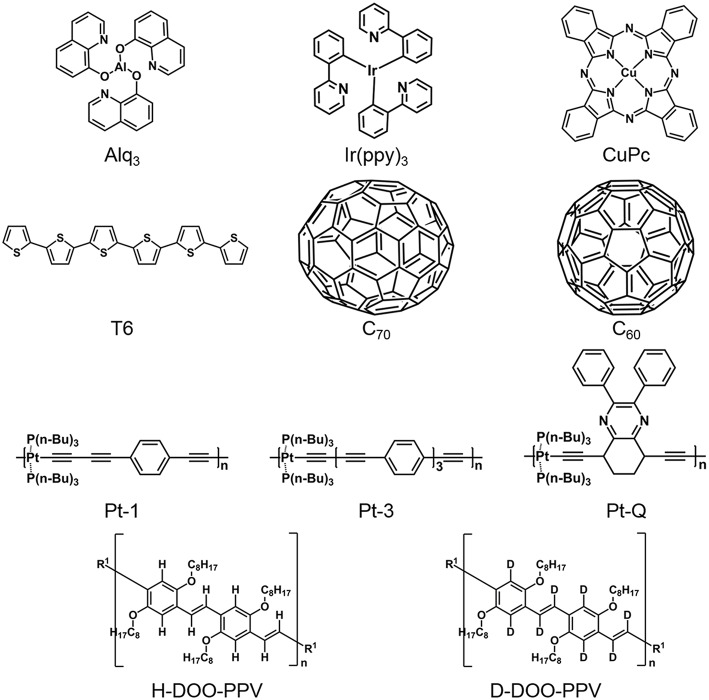
Chemical structures of molecules that have been employed in recent studies regarding spin transport.

By comparing the SOC strength of tris-(8-hydroxyquinoline) aluminum (Alq_3_) and tris(2-phenylpyridine) (Ir(ppy)_3_) whose chemical structures are similar, Wohlgenannt's group demonstrated that heavier atoms of Ir leads to a stronger SOC in the Ir(ppy)_3_ molecule (Figure 2) (Nguyen et al., 2007; Sheng et al., 2007). Furthermore, a series of molecules resembling such a molecular structure, Xq_3_ (X = Al, Ga, In, Bi) and triethylsilylethynyl (TES) series molecules, are studied through muon spin resonance (μSR) and intersystem crossing rates, where a similar dependence on the atom number has been observed (Nuccio et al., 2013). In addition to attributing the SOC strength to different elements, benefiting from the chemical designability of OSCs, the SOC strength can be modulated by synthesizing molecules containing different concentrations of heavy metal atoms. With an increased Pt concentration in three synthetic Pt-containing polymers, the SOC strength obviously increased, which can be found, on one hand, in the molecular structure (Pt concentration, Pt-1<Pt-3<Pt-Q, Figure 2). The distance between two adjacent Pt-atoms shrunk gradually with an increased Pt-atom concentration, and the intersystem-crossing (ISC) between the singlet and triplet states enhanced due to the localized Metal-to-Ligand Charge Transfer (MLCT), finally leading to an increased ratio of phosphorescence(Ph)/fluorescence(FL) (Sheng et al., 2013). On the other hand, from the measured results of inverse spin Hall voltage (V_ISHE_), the observation of the measured stronger SOC strength, corresponding to larger V_ISHE_, is consistent with the detected ratio of Ph/FL (Sun D. et al., 2016), which suggests the effect of atomic numbers.

Particularly, though theoretical calculations, Yu found that the SOC strength in sexythiophene (T6) and copper phthalocyanine (CuPc) was obviously lower than that in Alq_3_ molecule, although the atom weight of sulfur (S) and copper (Cu) elements were higher than the aluminum (Al) element. It was found that, T6 and CuPc are planar structures, whereas Alq_3_ is mainly a spatial stereostructure with three ligands arranged orthogonally, which enhances the spin mixing and thus leads to a stronger SOC (Yu, 2011, 2012). This report reveals that the SOC strength is not only related to the atomic number of materials but is also strongly affected by the molecular structure. The influence of the torsion angles of adjacent planar units in P3HT has also been researched through experiments and calculations, wherein, the large thiophene-thiophene dihedral angle corresponded to a larger spin admixture parameter which reflects the SOC strength, and thus a shorter spin diffusion length (Wang et al., 2019). Similarly, has also been found that the C_60_ expresses a large SOC even if it is purely composed by carbon atoms. Experimentally, through a more direct SOC measuring method of inverse spin Hall effect (ISHE), Sun et al. found that the SOC strength of C_60_ is even larger than the Pt-containing polymers. For such unusual observations, Sun et al. and Yu proved that the strong curvature of 60 carbon atoms on a spherical π orbitals results in the mixing of π and σ electrons (Yu, 2012; Sun D. et al., 2016). Moreover, with respect to the effect of curvature degree on the strength of SOC in fullerene-based materials, the effective SOC of C_60_ and C_70_ were estimated indirectly by obtaining the spin diffusion length in C_60_ film and C_70_ film by fitting the thickness dependence of magnetoresistance (MR) in OSVs using the modified Jullière equation, as well as the effective spin polarization at the interface of Co/C_60_ and Co/C_70_ using a first principle calculation. Stronger SOC in C_60_ and a higher spin polarization at Co/C_60_ interface, but shorter spin diffusion length, have been demonstrated, which reveals that the apparently larger SOC in C_60_ is derived from the lager curvature of the molecular structure (Liang et al., 2016).

### Hyperfine Interaction

HFI is another main intrinsic effect that can cause spin relaxation in OSCs, which primarily originates from the half-integer nuclear spins of atoms, for example, ^1^H, ^19^F, ^27^Al, ^63^Cu, etc. The half-integer nuclear spins leads to random magnetic fields which can indirectly couple to the spins of the carbon π electrons through the exchange interaction of carbon s electrons. Such interactions leads to the precession of the π electron spin, and consequently gives rise to spin relaxation (Bobbert, 2010; Yu et al., 2013).

Quantitative research, for HFI fields in a few molecules frequently employed in studies of spintronics, has already been implemented via first-principle calculations or ab initio calculations (Filidou et al., 2012; Giro et al., 2013; Yu et al., 2013). An effective HFI field for electron and hole polarons in molecular materials with H and deuterium(D)-substituted, have been shown in Yu's research (Yu et al., 2013). It has been found that substituting a given nucleus with its isotope, is an effective method to modify the HFI strength. And by replacing H by D via deuteration, the effective HFI field is expected to be reduced by a quarter in poly(dioctyloxy)phenylenevinylene (DOO-PPV) (Yu et al., 2013). Furthermore, relevant experimental evidence has also been reported by Vardeny's group (Nguyen et al., 2010b). The important role that HFI plays in spin transport has been demonstrated in a designed D-polymeric semiconductor where all ^1^H near the backbone carbon atoms on the polymer chains have been exchanged to ^2^H (D) atoms, from which the electronic properties are reserved but the nuclear magnetic moment becomes smaller. All devices based on the D-polymer have a larger spin valve effect and longer spin diffusion length than those based on the H-polymer, which is in agreement with the increase of the spin-relaxation time they measured. Besides deuteration, the substitution of ^12^C atoms with ^13^C atoms in the chemical backbone of the DOO-PPV polymers has also been studied, and the HFI increases with the half-integer nuclear spins of ^13^C, as expected (Nguyen et al., 2010a, 2011). Fullerene (C_60_) is considered to be a particularly unique molecule that only contains ^12^C and thus is considered to contain no HFI field. The HFI in C_60_ is estimated via organic magnetoresistance (OMAR) whose intrinsic mechanism can supposedly, according to some researchers, be interpreted by HFI (Kalinowski et al., 2003; Bobbert et al., 2007; Nguyen et al., 2007; Koopmans et al., 2011). Experimentally, no measurable OMAR effect is observed in C_60_-based devices, therefore, they note that the absence of hydrogen in most inorganic materials may be the reason why the OMAR effect cannot be found in inorganic spin devices. From the research of spin transport and OMAR (Bobbert, 2010), on one hand, HFI should be as small as possible to achieve a long spin lifetime and transport distance. On the other hand, HFI is considered to be larger due to its effect on OMAR (Sheng et al., 2006; McCamey et al., 2010; Nguyen et al., 2010a) and the manipulation of spins in molecular spintronics (Bobbert, 2014).

## Effect of Mobility on Spin Transport

Charge carrier mobility is another important factor that determines the spin transport process in OSCs. At the initial research stage, thin-film semiconductors with low mobility are usually used as the spin transport layer, which is normally prepared via thermal evaporation (Xiong et al., 2004; Nguyen et al., 2012). So far, although many significant contributions have already been made based on such thin films (Nguyen et al., 2012; Sun et al., 2017), the low mobility is clearly a bottleneck for further enhancing the spin transport performance. Thanks to the great development in the field of organic electronics, especially the prosperous research on organic field effect transistors (OFETs) in the past decades, many high-mobility organic semiconductor materials have been developed (Dong et al., 2013; Gao and Zhao, 2015), which provides strong material support for the current research of spin transport. For instance, the high mobility n-type semiconductor, poly[N,N′-bis(2-octyldodecyl)-1,4,5,8-naphthalenedicarboximide)-2,6-diyl]-alt-5,5′-(2,2′-bithio-phene) [P(NDI2OD-T2)] with the electron mobility of 0.2~0.85 cm^2^/Vs, has been employed to build OSVs and a very long spin transport distance of 64 nm has been observed at 4.2 K (Li et al., 2015). The OSC single crystal has long been considered to be the ideal material for spin transport, since it possesses both a long spin relaxation time and very high mobility due to the relatively pure matrix and thus weak scattering during the transport process of spin/charge carriers (Wang et al., 2018; Zhang et al., 2018). Additionally, supramolecular semiconductors, especially the cocrystals, have also exhibited significant advantages in terms of high conductivity, photoelectric property and non-linear optics, etc (Zhu et al., 2015; Wang et al., 2016). With the target of both high mobility for long spin transport distance and multifunctional applications, single crystal, and supramolecules are very promising for the future development of organic spintronics, however related research is currently still in its infancy.

### OSC Thin Films

Since the first observation of giant magnetoresistance (GMR) in 8-hydroxy-quinoline aluminum (Alq_3_) (Xiong et al., 2004), a variety of OSC thin films have been applied as the spacer in spin valves. Table 1 shows a summary of the spin transport distances and corresponding mobilities of few OSCs that have been widely employed in previous studies of spintronics. Although the experimentally measured spin transport distance is comprehensively affected by magnetic electrodes, spin injection efficiency, device fabrication, etc (Dediu et al., 2009), a clear relationship that can be found is that the spin transport distance has obviously been enhanced in OSCs with higher mobility. Additionally, it worth noting from Table 1 that a high-mobility polymer, known as P(NDI2OD-T2), corresponds to a relatively short spin transport distance of 42 nm at 300 K, which can be attributed to the vacancy or solvent residue in P(NDI2OD-T2) film prepared via the solution method (Li et al., 2015), whereas other organic semiconductors in Table 1 are prepared via thermal evaporation. In fact, the defects in organic molecules will lead to strong spin scattering centers which are much more sensitive to defects in contrast to the carrier mobility of thin films. With regard to defects induced via the solution method, Mei's group have developed a novel melt-processing method which can clearly decrease the defect density and thus achieve an excellent charge carrier transport in thin films (Zhao et al., 2017, 2018).

**Table 1:** Mobility of thin-film OSCs and the corresponding spin transport distance in spintronic devices.

**Organic semiconductor**	**Mobility of thin film (cm^**2**^/Vs)**	**Device structure (bottom to top)**	**Spin transport distance**
Alq_3_	2 × 10^−8^~2 × 10^−10^ (Chen et al., 1999)	Co/Al_2_O_3_/**Alq**_**3**_/Ni_80_Fe_20_	1.6 nm @ 300 K (Santos et al., 2007)
		Ni/**Alq**_**3**_/Co	4.25 nm @ 50 K (Pramanik et al., 2007)
		LSMO/**Alq**_**3**_/Co/Al	45 nm @ 11 K (Xiong et al., 2004)
Rubrene (Amorphous)	~10^−6^ (Seo et al., 2006)	Fe_3_O_4_/AlO/**Rubrene**/Co	20 nm @ 300 K (Zhang et al., 2015)
		Fe/**Rubrene**/V[TCNE]_X_/Al	10 nm @ 300 K (Li et al., 2011)
		Fe/Al_2_O_3_/**Rubrene**/Co/SiO	13.3 nm @ 0.45 K(Shim et al., 2008)
BCP	5 × 10^−6^ (Liu et al., 2011)	Co/leaky AlOx/**BCP**/Ni_80_Fe_20_	60 nm @ 300 K (Sun et al., 2013)
C_60_ (Amorphous)	1.4 × 10^−5^ (Im et al., 2011)	MgO/Fe_3_O_4_/Al-O/**C**_**60**_**/**Co/Al	110 nm @ 300 K (Zhang J. et al., 2013)
		LSMO/**C**_**60**_/AlOx/Co	36 nm @ 300 K (Li et al., 2014)
		LSMO/**C**_**60**_/Co/Al	12 nm @ 10 K (Nguyen et al., 2013)
F_16_CuPc	9 × 10^−4^ (Sun X. et al., 2016)	Co/AlOx/**F**_**16**_**CuPc**/Ni_80_Fe_20_	180 nm @ 300 K (Sun X. et al., 2016)
P(NDI2OD-T2)	0.2~0.85 (Yan et al., 2009)	LSMO/**P(NDI2OD-T2)**/AlOx/Co	42 nm @ 300 K (Li et al., 2015)
			64 nm @ 4.2 K (Li et al., 2015)

A number of novel high-mobility OSCs (>5 cm^2^/Vs) and feasible methods for achieving high-performance charge carrier transport have also been summarized in recent reviews (Tsao and Mullen, 2010; Dong et al., 2013; Gao and Zhao, 2015), and herein, several important points for selecting high-mobility semiconductors used in organic spintronic field were extracted. First, the condensed π-π stacking and little tilt angle of adjacent molecules will help increase the charge transfer integrals and thus the mobility, which can be realized via appropriate molecular design engineering, such as maximizing the intermolecular π-conjugated and by introducing intermolecular hydrogen-bonding or dipole-dipole interaction, and by increasing the intrachain conjugated length, etc. Second, morphology control is another key point for obtaining high mobility, and an accepted fact is that polycrystalline or ordered OSCs possess a higher mobility than a disordered structure. A grain boundary separating the packed crystalline domains is the main obstacle for pursing high mobility, and it can be highly reduced by controlling the chemical structure and molecular weight design, solvent or thermal annealing, etc. For simply fabricating an ordered thin film, a novel method of using Chinese brushes to control liquid transfer can make the molecules more ordered along the brushing direction and thus leading to an enhanced electrical property (Lin et al., 2017).

**Figure 3:**
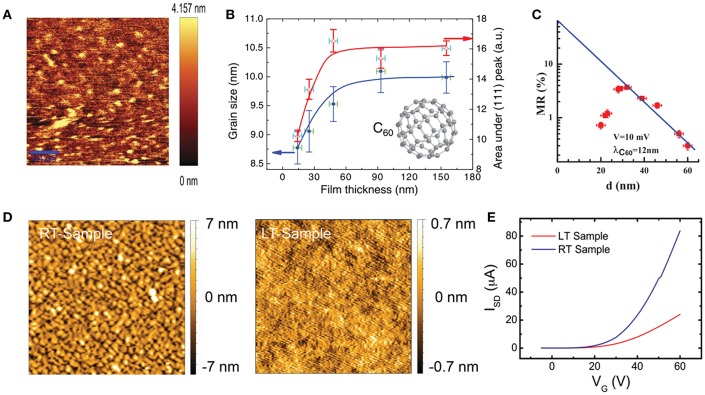
The effect of morphology on carrier mobility and spin transport distance. AFM image of C_60_ thin film **(A)**. The relationship between grain size and film thickness, and the area of (111) Bragg scattering peak and film thickness **(B)**. C_60_ thickness dependence of MR with bias of 10 mV **(C)**. [A&B&C reproduced from (Nguyen et al., 2013), with permission of American Physical Society] AFM images of F_16_CuPc fabricated at RT and LT **(D)**. Transfer curves of F_16_CuPc-based OFET at RT and LT **(E)**. [D&E reproduced from (Sun X. et al., 2016), with permission of John Wiley and Sons].

In previous studies it has been found that the grain boundaries can also work as the spin scattering centers in polycrystalline OSC thin films. The correlation between grain boundary density and spin transport performance has been investigated in polycrystalline C_60_ thin films whose carrier mobility can be changed from 2 to 5 cm^2^/Vs according to the controllable grain size (Kwiatkowski et al., 2009). Recently, Nguyen et al. prepared C_60_ thin films with grain sizes between 8.8 and 10.5 nm by simply controlling the grown thicknesses of the films (Figures 3A,B) (Nguyen et al., 2013). It was found that the spin transport performance decreases with the increased thickness of the molecular layer, nevertheless, a variation of GMR has been measured and the highest MR is measured at the thickness of 45 nm (Figure 3C). Thus, it is clear that the dominant effect on spin transport distance has been changed from the mobility to morphology effect enhanced by spin scattering along with the increased thickness, which means the gradually increased grain boundary density in the film enhances the surface spin-flip probability and therefore weakens the spin transport distance. Similar morphology effects on carrier/spin transport performance have also been observed in other studies (Shim et al., 2008; Bobbert et al., 2009). Recently, Sun et al. demonstrated that the homogeneous amorphous OSC thin films may possess much better spin transport performance by excluding the negative effect of grain boundary density in thin-film spin valves (Table 1) (Sun X. et al., 2016). In that study, polycrystalline F_16_CuPc has shown distinctive grain boundaries and much larger root-mean-square (RMS) roughness than the amorphous one (Figures 3D,E). Although the amorphous F_16_CuPc deposited at low temperature (LT) merely shows a mobility of 0.9 × 10^−3^ cm^2^/Vs, much lower than the polycrystalline one (4.2 × 10^−3^ cm^2^/Vs), a large spin diffusion length up to 180 nm has been observed at room temperature (RT) which, to our knowledge, is the best recorded molecular matrix so far. According to the above studies, OSC materials with high mobility and weak spin scattering factors are very promising for spin transport applications. In fact, a long spin transport distance up to hundreds of nanometers or even at the millimeter level in single-crystal rubrene has been predicted in a theoretical study (Yu, 2012), whereas the amorphous rubrene thin film has shown a spin diffusion length of merely 13.3 nm (Shim et al., 2008).

### Single Crystals

Organic semiconductor single crystals, with a perfect crystal texture, minimal density of defects, and an absence of grain boundaries have exhibited very high mobility (>5 cm^2^/Vs) and unique physicochemical properties, which have developed rapidly in recent years (Briseno et al., 2006; Fan et al., 2013; Wang et al., 2018; Zhang et al., 2018). Different from the hopping transport mode of carriers in thin-film OSCs, delocalized band-like charge transport is presented in organic single crystals, which possesses a negative temperature coefficient of mobility (Krupskaya et al., 2015; Xu et al., 2016). The long range ordered arrangement of the molecules leads to anisotropic charge-transport properties and carrier mobility. Generally, large π-stacking and strong transfer integrals will lead to an efficient charge transport along the π-π stacking direction (Zhang et al., 2018), and if the current direction is perpendicular to the strong interlayer electronic couplings, the charge transport will be diminished remarkably (Zhang Z. P. et al., 2016), which means that the called “face on” stacking style will be beneficial for the vertical spin devices and the “edge on” style will be more beneficial for lateral spintronic devices. However, the effect of stacking style on spin transport performance has not been systematically studied so far, and more research efforts still need to be devoted on this point. In a recent report, Tsurumi et al. studied the spin transport process and relaxation mechanism in a lateral-structured device based on C_10_-DNBDT-NW single crystals, where the Hall mobility reached 16.5 cm^2^/Vs at the in-plane direction, while the experimental measured spin relaxation time reached 16.6 ns at RT. According to the Einstein relationship, the corresponding spin transport distance is estimated to be 840 nm at RT and up to 1.6 μm at 50 K, which shows the great potential of single crystals in the application of spintronic devices (Tsurumi et al., 2017). However, this experimental lateral device does not have a real spin injection process, which means it is not a true sense of the spintronic device and also implies a big challenge in using single crystals within the research field of spintronics.

**Figure 4:**
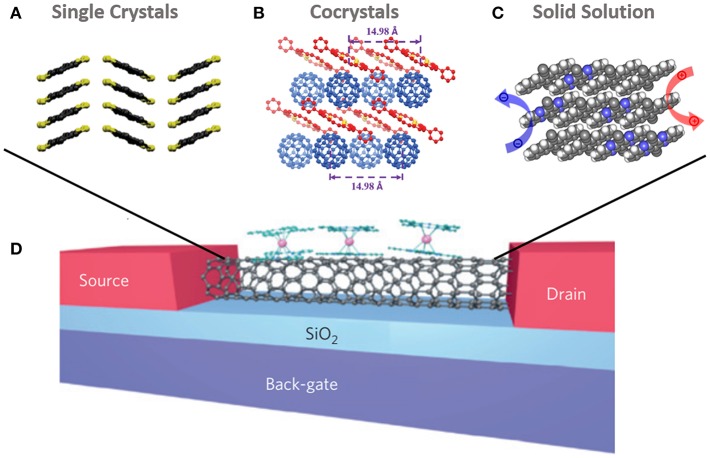
Schematic diagram of lateral spintronic devices based on single crystals [Reproduced from Zhang et al., 2016, with permission of John Wiley and Sons] **(A)**, cocrystals [Reproduced from Zhang et al., 2016, with permission of John Wiley and Sons] **(B)** and solid solutions [Reproduced from (Xu et al., 2015), with permission of American Chemical Society] **(C)**. Schematic diagram of a supramolecular spin valve based on SWCNT, coupled with modified TbPc_2_ single-molecule magnets [Reproduced from (Urdampilleta et al., 2011), with permission of Springer Nature] **(D)**, where the supramolecules of SWCNT coupled with modified TbPc_2_ can be substituted by single crystals, cocrystals, and solid solutions.

As for using single crystals to fabricate spintronic devices and research spin transport properties, several challenges need to be addressed or paid attention to. With regard to spintronic device preparation, the fabrication of the top magnetic electrode is a serious issue, since the organic single crystal tends to be damaged by the very high temperature during the process (Sun et al., 2014). So far, the buffer layer assisted deposition (BLAG) (Sun et al., 2010) and the liquid nitrogen cooling method (Sun et al., 2013) are demonstrated to be very promising in avoiding metal penetration in organic spintronic devices during top electrode deposition on OSC thin films. However, such methods have not yet been applied to fabricate electrical devices based on a single crystal. For fabricating single-crystal-based electronic devices such as OFETs, thermal evaporation at a high vacuum and stamping Au layer are usually employed to pattern the source and drain electrodes on OSC crystals (Zhang et al., 2010; Kikuchi et al., 2017). Similar methods may possibly also be introduced for fabricating single crystal based spintronic devices by replacing the Au with magnetic metals, however, there are of course many technical challenges that still need to be overcome. Particularly, with regard to lateral single-crystal spintronic devices (Figure 4A), the limitation of existing spin transport distance will be an issue, since the longest spin transport distance is merely 180 nm currently (Sun X. et al., 2016; Tsurumi et al., 2017). In addition, spin injection, a key point for successfully detecting spin signals, may suffer from the influence of bad contact between the magnetic electrode and single crystal, which is limited by both the dedicated device structure and the current level of lab-scale fabrication techniques. Indeed, spintronic devices based on an organic single crystal is a novel research field and shows abundant possibilities due to its unique properties, however, various issues need to be addressed in the exploration of this new direction.

### Supramolecules

The π-conjugated supramolecular materials are normally assembled by two or more kinds molecules that held together by non-covalent intramolecular interactions, such as π-π stacking, hydrogen-bonding and dipole-dipole interactions etc (Ghosh et al., 2017; Ikeda and Haino, 2017), which generally exhibit a special functionality compared to homogeneous OSC and have shown great potential in functional spintronic devices. Based on supramolecular material, Urdampilleta et al. fabricate a lateral spintronic device, in which the source and drain electrodes are palladium and the charge transport layer is composed of a single-walled carbon nanotube (SWCNT) coupled with modified TbPc_2_ single-molecule magnets (Pc = phthalocyanine) through supramolecular π-π interaction (Figure 4D). When non-spin-polarized carriers transport in SWCNT, the localized magnetic moments from TbPc_2_ will lead to obvious magnetic field dependence of conductance, where a magnetoresistance up to 300% can be observed below 1 K (Urdampilleta et al., 2011). Although this supramolecular spin valve has not shown a classical spin transport process in the molecular matrix, it still presents a very promising future of supramolecular assemblies for spintronic applications.

More functional supramolecular semiconductor materials are eagerly expected to be applied in the study of spin transport and functional spintronic devices, especially after the success of the first molecular spin photovoltaic (MSP) device, in which the spin transport, coupled with a photovoltaic effect, has been firstly achieved in molecule-based devices (Sun et al., 2017). However, in this study, the MSP device just shows an extremely low power conversion efficiency (PCE) since the active layer consists of only one component C_60_, which is clearly a large limitation for further enhancing the spin performance of this device. As known in the organic photovoltaic (OPV) field, bulk heterojunction structured devices hold much higher PCE than single layer devices, where the supramolecular interaction and assembly of donor and accepter materials plays a crucial role (Wurthner, 2016; Ghosh et al., 2017). The purpose of studying supramolecular assembly in OPV is mainly to obtain a high charge transfer rate and low charge recombination in the perpendicular direction, which is partially consistent with the demand of vertical spintronic devices. However, the interface induced by a two-phase structure may enhance the spin scattering, which is similar to the impact of grain boundaries. Therefore, how to utilize the supramolecular Nano assembly to modulate the microphase separation, and thus to reduce spin scattering and enhance PCE, will be a very interesting research direction in the future.

Organic cocrystals, composed of two or more components through supramolecular interaction, is a single-phase semiconductor and appears to exhibit more benefits for spin transport (Zhu et al., 2015; Wang et al., 2016), in which the defect induced charge/spin carrier scattering can be minimized (Figure 4B). Hydrogen-bonded, halogen-bonded, π-π stacking and charge transfer are the four typical supramolecular interactions in organic cocrystals. The charge transfer interaction is an especially important type, showing high conductivity (Hiraoka et al., 2007; Zheng et al., 2018), while the π-π stacking interaction tends to make a contribution to the photoelectronic properties of cocrystals (Zhang J. et al., 2013). At present, organic cocrystals have already been preliminarily used in lateral OPV devices. Even though a PCE of only 0.27% has been obtained based on C_60_-DPTTA with the electron and hole mobility of 0.01 and 0.3 cm^2^/Vs, respectively, it distinctly demonstrates the possibility for applying cocrystal in MSP devices (Zhang et al., 2016b). In addition, solid solutions and doped crystals are two novel types of supramolecular crystals which also present unique photoelectric properties and high conductivity (Figure 4C). The different two-phase proportions in cocrystals, solid solutions and doped crystals may lead to different spin scattering strengths and spin transport performances, certainly it needs sufficient investigations in the future. Once the supramolecular semiconductors are successfully applied in spintronics, both the interesting functions and attractive transport properties in supramolecules should enrich the theoretical and experimental research of this field.

## Conclusion and Outlook

Spin transport greatly depends on the spin relaxation time and carrier mobility of OSCs, where elementary composition, molecular structure, packing mode, aggregation structure, and morphology play crucial roles. In this review, correlations between the above parameters and spin transport performance are systemically discussed, and some primary guidelines for enhancing spin transport performances in π-conjugated molecular materials are provided. SOC and HFI, as two principle spin relaxation mechanisms, are closely related to elementary composition and molecular structure, which can be distinctly weakened by modifications on the molecular structure. In addition, on the basis of effectively controlling the spin scattering which originates from grain boundaries and other structure defects, the high mobility of π-conjugated molecular materials is demonstrated to be very promising for long-distance spin transport. Particularly, the ordered stacked aggregation of molecules with both high mobility and weak spin scattering factors possess great potential in high-performance spin transport, including single crystals and supramolecular Nano assembled structures (especially organic cocrystals). In terms of current developments in organic electronics and spintronics, spintronic devices based on single crystals or cocrystals can only be applied through lateral device structures, and many restrictions at the technical level still need to be overcome, while reliable device fabrication and efficient spin injection should receive more attention in the future.

## Author Contributions

LG and YQ co-wrote the paper. XG completed the spelling and grammar check, and copyright section. XZ and XS supervised this review and completed all the submissions. All authors joined the discussion and revision of this paper.

### Conflict of Interest Statement

The authors declare that the research was conducted in the absence of any commercial or financial relationships that could be construed as a potential conflict of interest.
